# STN7 Operates in Retrograde Signaling through Controlling Redox Balance in the Electron Transfer Chain

**DOI:** 10.3389/fpls.2012.00277

**Published:** 2012-12-19

**Authors:** Mikko Tikkanen, Peter J. Gollan, Marjaana Suorsa, Saijaliisa Kangasjärvi, Eva-Mari Aro

**Affiliations:** ^1^Molecular Plant Biology, Department of Biochemistry and Food Chemistry, University of TurkuTurku, Finland

**Keywords:** STN7, redox, thylakoid, retrograde signaling, reactive oxygen species, jasmonate biosynthesis and signaling

## Abstract

Phosphorylation of the major photosynthetic light harvesting antenna proteins by STN7 kinase balances excitation between PSII and PSI. Phosphorylation of such abundant proteins is unique, differing distinctively from conventional tasks of protein kinases in phosphorylation of low abundance proteins in signaling cascades. Excitation balance between PSII and PSI is critical for redox homeostasis between the plastoquinone and plastocyanin pools and PSI electron acceptors, determining the capacity of the thylakoid membrane to produce reactive oxygen species (ROS) that operate as signals relaying information between chloroplasts and other cellular compartments. STN7 has also been proposed to be a conventional signaling kinase, instigating the phosphorylation cascade required for coordinated expression of photosynthesis genes and assembly of the photosynthetic machinery. The absence of STN7 kinase, however, does not prevent plants from sensing redox imbalance and adjusting the stoichiometry of the photosynthetic machinery to restore redox homeostasis. This suggests that STN7 is not essential for signaling between the chloroplast and the nucleus. Here we discuss the evolution and functions of the STN7 and other thylakoid protein kinases and phosphatases, and the inherent difficulties in analyzing signaling cascades initiated from the photosynthetic machinery. Based on our analyses of literature and publicly available expression data, we conclude that STN7 exerts it signaling effect primarily by controlling chloroplast ROS homeostasis through maintaining steady-state phosphorylation of the light harvesting II proteins and the redox balance in the thylakoid membrane. ROS are important signaling molecules with a direct effect on the development of jasmonate, which in turn relays information out from the chloroplast. We propose that thylakoid membrane redox homeostasis, regulated by SNT7, sends cell-wide signals that reprogram the entire hormonal network in the cell.

## Introduction

Reversible protein phosphorylation modulates the cellular activity of target proteins by altering their chemical and structural characteristics, and is a ubiquitous mechanism for transmitting signals throughout the cell. Phosphorylation signal cascades usually involve the sequential phosphorylation and/or dephosphorylation of low abundance proteins including kinases, phosphatases, and protein receptors, culminating in the regulation of countless cellular metabolic processes. The reversible phosphorylation of several proteins in the plant thylakoid membrane is atypical, as these are among the most abundant membrane proteins in nature, and the physiological importance of this phenomenon is not yet fully understood. Phosphorylation of thylakoid proteins is known to play a role in coordinating the assembly and repair of the photosynthetic machinery, and in maintaining redox balance in the photosynthetic electron transfer chain (ETC) under changing environmental conditions. Membrane protein phosphorylation is also implicated in the transmission of retrograde signals from the thylakoid in response to the redox state of the intersystem ETC. This phosphorylation-mediated signal cascade has been suggested as a means of regulating the expression of chloroplast- and nuclear-encoded photosynthetic genes in response to environmental cues, hypothetically linking the operational status of the photosynthetic machinery with its assembly, repair, and acclimation according to environmental cues. The existence of such a retrograde mechanism remains hypothetical and any involvement of membrane phosphorylation, either directly or indirectly, is unclear.

At least 15 kinases and 10 phosphatases exist in higher plant chloroplasts (Bayer et al., 2012), but so far only the kinases STN7 (Bellafiore et al., 2005) and STN8 (Bonardi et al., 2005) and the phosphatases TAP38 (Pribil et al., 2010), also called PPH1 (Shapiguzov et al., 2010), and PBCP (Samol et al., 2012) have demonstrated their involvement in the heavy-duty phosphorylation of thylakoid proteins. The exploration of a possible retrograde signaling network involving thylakoid membrane phosphorylation has largely concentrated on the activities of these kinases and phosphatases, particularly on the biophysical characterizations of knockout mutants (Bellafiore et al., 2005; Bonardi et al., 2005; Pribil et al., 2010; Shapiguzov et al., 2010; Tikkanen et al., 2010; Samol et al., 2012). However, deciphering the results of these experiments is often complex due to the primary roles of STN7, STN8, TAP38, and PBCP in maintaining redox homeostasis and proper assembly of thylakoid protein complexes, as well as the existence of multiple redox signaling pathways extending from the chloroplast. In this review we discuss the possible roles of membrane protein phosphorylation in retrograde signaling in the context of recent discoveries and common misconceptions in this field. The importance of STN7, STN8, TAP38, and PBCP in the photosynthesis, stress response, and plant development are examined in light of our *in silico* analysis of their expression and evolution in plants. Our analysis describes the far-reaching impact of thylakoid protein phosphorylation on cell signaling, plant growth, and stress response that arises from a primary role of the kinases/phosphatases in safeguarding photosynthetic redox homeostasis and production of reactive oxygen species (ROS).

## Thylakoid Protein Phosphorylation; The Known and the Unknown

The importance of reversible LHCII protein phosphorylation in inducing state transitions in the photosynthetic machinery is well known (Allen et al., 1981; Depege et al., 2003; Bellafiore et al., 2005; Pribil et al., 2010; Shapiguzov et al., 2010), but only recently it was established that the *steady-state* phosphorylation of PSII and LHCII proteins ensures balanced distribution of energy to both photosystems to control the redox status of the ETC (Grieco et al., 2012). The STN7 kinase is responsible for phosphorylation of LCHII proteins and, to a lesser extent, the PSII core proteins CP43, D1, and D2 (Bellafiore et al., 2005; Fristedt and Vener, 2011), while STN8 primarily targets the PSII core proteins (Bonardi et al., 2005; Vainonen et al., 2005) and exclusively phosphorylates the calcium signaling membrane protein Calcium Sensing Receptor (CAS; Vainonen et al., 2008). Dephosphorylation of LHCII proteins is carried out predominantly by the TAP38/PPH1 phosphatase (Pribil et al., 2010; Shapiguzov et al., 2010), while the PBCP phosphatase operates mainly in PSII core dephosphorylation (Samol et al., 2012); however, like the kinases, these two phosphatases also show some substrate overlap (Samol et al., 2012). STN7-mediated LHCII phosphorylation is required to provide sufficient excitation energy to PSI in order to maintain excitation and functional balance of PSII and PSI, which is especially important under low levels of white light, where the thermal dissipation of excitation energy is low and energy transfer from LHCII to the photosystems must be efficient (Finazzi et al., 2004; Tikkanen et al., 2010, 2011). In contrast, strong STN7- and STN8-dependent reversible phosphorylation of thylakoid proteins, induced by extreme changes in low-intensity light quality that preferentially excites either PSI (far red light) or PSII (red and blue light), distorts the excitation balance between PSII and PSI and leads to “state transitions.”

In addition to its role in directly maintaining excitation balance within the photosynthetic system under white light conditions, the STN7 kinase itself is also implicated in retrograde signaling from the chloroplast to the nucleus in order to regulate the expression of nuclear-encoded photosynthetic proteins (Wagner et al., 2008; Bräutigam et al., 2009; Pesaresi et al., 2009, 2010; Leister, 2012). This so-called long-term response (LTR) occurs over a course of hours and days to readjust the stoichiometry of the components of the photosynthetic apparatus according to retrograde signals. STN7 in particular is anticipated to be an important instigator of redox-responsive retrograde signaling due to its redox-sensitive kinase activity (Pesaresi et al., 2009); however, the details of any such role and the identities of signaling intermediates are unknown. As such, the importance of STN7 and thylakoid phosphorylation for retrograde signaling remains speculative.

Analysis of *stn7* mutants in our lab shows that strong reduction of the intersystem ETC in the absence of STN7 leads to an increase in the PSI-to-PSII ratio during thylakoid membrane biogenesis (Tikkanen et al., 2006), which acts to restore the ETC redox balance and thus makes the *stn7* mutant behave like the wild type under constant growth condition (Tikkanen et al., 2006; Grieco et al., 2012). These results clearly show that *stn7* plants have the ability to monitor the redox balance of the ETC and can carry out retrograde signaling independently of any STN7-mediated phosphorylation. However, we did observe stunted growth and diminished relative amounts of PSI in *stn7* plants grown under normal growth light that was interrupted with high light peaks (Tikkanen et al., 2010), in line with the results obtained from plants that were treated with alternating 1 h of PSII and 1 h of PSI lights or with 1 h of 50 and 1 h of 240 μmol m^−2^ s^−1^ of light (Bellafiore et al., 2005), indicating that STN7 is required for effective retrograde signaling under these stressful conditions. As such, the possibility of a primary role for STN7 in phosphorylation-mediated retrograde signaling cannot be excluded, but the notion that STN7 *per se* is vital in this process appears to be untrue. This assertion challenges the *status quo* and is currently rather contentious, but the conclusion that STN7 is central to redox-induced LTR based on manipulation of the intersystem ETC in *stn7* mutants using of distinct light qualities (Bräutigam et al., 2009) may be misleading. The change in gene expression can be largely explained by the incapability of *stn7* to oxidize the ETC due to poor capture of photons by PSI in the absence of LHCII phosphorylation. Indeed, comparing *stn7* with various PSI subunit mutants that have functional STN7 kinase and LHCII phosphorylation, but are differently inhibited in the transfer of energy from P-LHCII to PSI, reveals varying levels of ETC redox unbalance that can easily account for the slight differences in the levels of gene expression, the majority of which follow a common trend among these mutants. It should also be noted that *stn7* grown in white light prior to treatment with PSI and PSII lights has corrected redox imbalance by increasing the amount of PSI complexes. However, these accumulated PSI centers become useless in PSII light, which is hardly absorbed by PSI, leading to ETC imbalance and altered redox signaling in *stn7*. Conversely, under far red light where LHCII is dephosphorylated in both WT and *stn7*, the abnormally high quantity of PSI in *stn7* is fully operational and efficiently oxidizes the ETC, again deviating the redox signaling of *stn7* from that of WT. It is important to recognize the difficulty in dissecting the effects of altered redox states in chloroplasts from those due the specific absence of STN7 in mutant plants, and these results must be interpreted carefully.

The main role of STN8-mediated phosphorylation of the PSII core proteins appears to involve the regulation of fluent turnover of PSII, although STN7 also takes part in this process (Vainonen et al., 2005; Fristedt and Vener, 2011; Tikkanen and Aro, 2011). A further role of thylakoid protein phosphorylation exists in coordinating the assembly of the photosynthetic complexes. Phosphorylation of subunits in photosynthetic protein complexes contributes negative charges that are required for the correct structure of the machinery (Barber, 1980; Kruse et al., 1997), which is particularly important for fluent turnover of PSII under high light intensity (Tikkanen et al., 2008) and is also likely to play a part in prompt synthesis of the thylakoid protein complexes during rapid growth of new green tissues. The synthesis and assembly of the photosynthetic complexes proceed in a highly coordinated and stepwise process that is constantly regulated by dozens of proteins through feed-back mechanisms from previous assembly steps (Mulo et al., 2008). Tight regulation of this process is vital for synchronized nuclear and chloroplast gene expression, and to control the biosynthesis and membrane incorporation of chlorophyll-protein complexes, which are photosensitive and can produce damaging ROS. The activities of STN7, STN8, TAP38, and PBCP in regulating thylakoid phosphorylation positions them as important regulators of assembly of the photosynthetic machinery, and it follows that membrane signals will be altered in mutants lacking these kinases and/or phosphatases, further complicating the study of their direct involvement in retrograde signaling.

## STN7 and STN8 Domain Structure and Evolution

**Figure 1:**
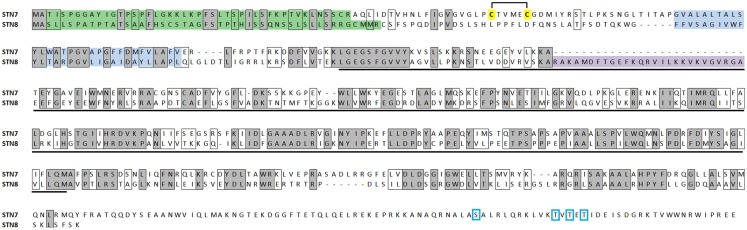
**Alignment of the amino acid sequences of STN7 (At1g68830) and STN8 (At5g01920) showing sites of identical (shaded, boxed) and similar (boxed) residues**. The signal peptides (predicted using ChloroP software; http://www.cbs.dtu.dk/services/ChloroP/) are shown in green, transmembrane domains (predicted using TMHMM; http://www.cbs.dtu.dk/services/TMHMM/) are shown in blue, Ser/Thr kinase domains (detected using NCBI Conserved Domain Search; http://www.ncbi.nlm.nih.gov/Structure/cdd/wrpsb.cgi) are underlined in black. A hydrophilic sequence unique to SNT8 is shown in purple, disulfide bridge-forming cysteines in SNT7 are highlighted yellow and phosphorylated residues are boxed in blue.

Duplication of an ancestor serine/threonine kinase gave rise to STN7 and STN8, which share over 30% amino acid identity in *Arabidopsis*. Each conserves the characteristic serine/threonine-targeting kinase domain as well as a hydrophobic transmembrane region that is responsible for their localization in the thylakoid membrane (Figure 1). Unlike STN8, STN7 possesses a pair of cysteine residues situated on the N-terminal of the transmembrane domain that form a lumen-exposed disulfide bridge that is thought to modulate its kinase activity (Lemeille et al., 2009), while a unique C-terminal domain contains four sites of reversible phosphorylation which regulate the accumulation of STN7 in the membrane (Willig et al., 2011). STN8 is distinct from its paralog in its inclusion of a charged region close to the N-terminus of the kinase domain that does not appear in STN7. These characteristic features are well conserved in orthologs of STN7 and STN8 in other plants (discussed below), suggesting that the roles for the thylakoid kinases are conserved across the plant kingdom.

**Figure 2:**
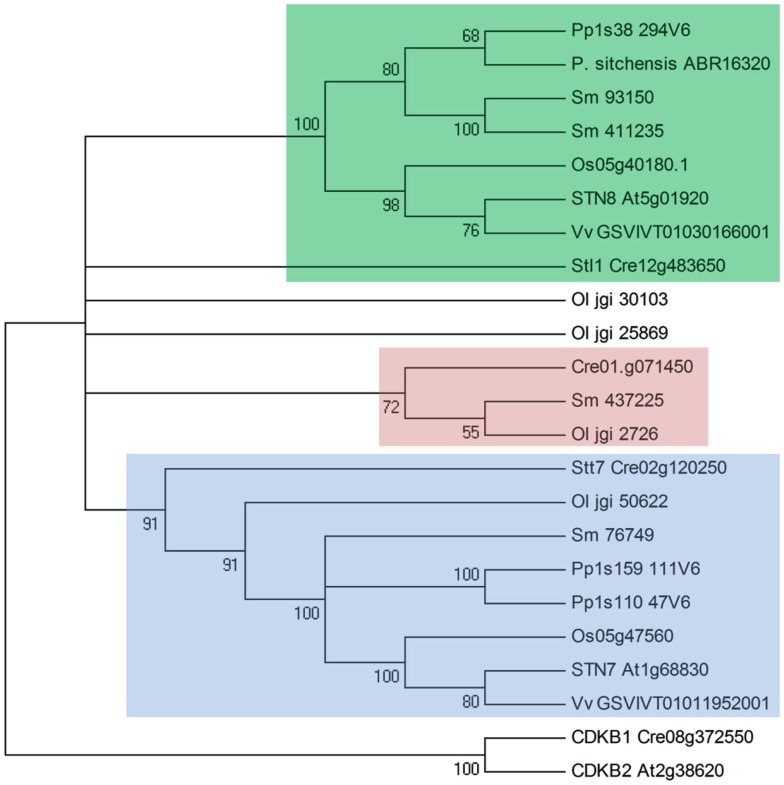
**Phylogenetic relationships between STN7, STN8, and their homologs among the green plant lineage**. Shown is a condensed Neighbor-Joining tree constructed from amino acid sequences using Poisson correction in MEGA5. Numbers indicate bootstrapping values for branching points. STN7 and orthologs are highlighted blue, STN8 and orthologs are highlighted green, and predicted mitochondrial duplications within the gene family are highlighted red. Protein sequences were retrieved from the Phytozome, JGI, and NCBI GenBank genome databases, with gene identifiers shown for *Arabidopsis thaliana* (At), *Oryza sativa* (Os), *Vitis vinifera* (Vv), *Physcomitrella patens* (Pp), *Picea sitchensis* (*P. sitchensis*), *Selaginella moellendorffii* (Sm), *Chlamydomonas reinhardtii* (Cre), and *Ostreococcus lucimarinus* (Ol). Cyclin-dependent kinase B (CDKB) sequences were included as the nearest plant homologs to the STN7/STN8 kinases.

Duplicate kinase orthologs of STN7 and STN8 were identified in all members of the green plant lineage examined in this work, with the exception of the conifers that appear to lack an STN7 homolog (Figure 2). The existence of homologs in the green algae *Chlamydomonas reinhardtii* (called Stt7 and Stl1, respectively) and *Ostreococcus lucimarinus* but not in red algae or diatoms (Grouneva et al., 2012), indicate that duplication of the original serine/threonine kinase took place in the early stages of green plant evolution. Although they are relative outliers among plant serine-threonine kinases, the isolated kinase domain sequences of STN7 and STN8 show considerable homology to the B-type cyclin-dependent kinases (CDKB), which are plant-specific members of the eukaryotic CDKs that have diverged from other eukaryotic CDKs, operating to regulate gene expression and cell differentiation exclusively in plants (Corellou et al., 2005). The functional motifs required for cyclin-binding and regulation of CDKB kinase activity are not conserved in STN7 or STN8, however the possibility that the heritage of these thylakoid kinases lies in the regulation of cell cycling suggests their cellular influence may extend beyond dynamic reorganization of the thylakoid membrane to a broader role in cell signaling.

Single, well conserved STN7 and STN8 orthologs occur in higher plants, while additional duplications within this group were identified in lower plants (Figure 2). *STN7* duplication has occurred independently and relatively recently in the moss *Physcomitrella patens*, with both descendant genes contributing transcripts that encode typical STN7 proteins. Interestingly *P. patens* also contains duplicate *TAP38* genes, perhaps indicating a development of two distinct antagonistic STN7/TAP38 systems regulating thylakoid membrane protein phosphorylation. The genome of the lycophyte *Selaginella moellendorffii* encodes two STN8-like kinases that are predicted to be chloroplast-localized, while *S. moellendorffii* and the algae *C. reinhardtii* and *O. lucimarinus* all contain duplications of STN7/STN8 genes that are predicted to be mitochondria-targeted and are not found in higher plants.

## Thylakoid Membrane Protein Kinase/Phosphatase Expression Analyses Using Microarray Data; Expression during Plant Growth and Development

**Figure 3:**
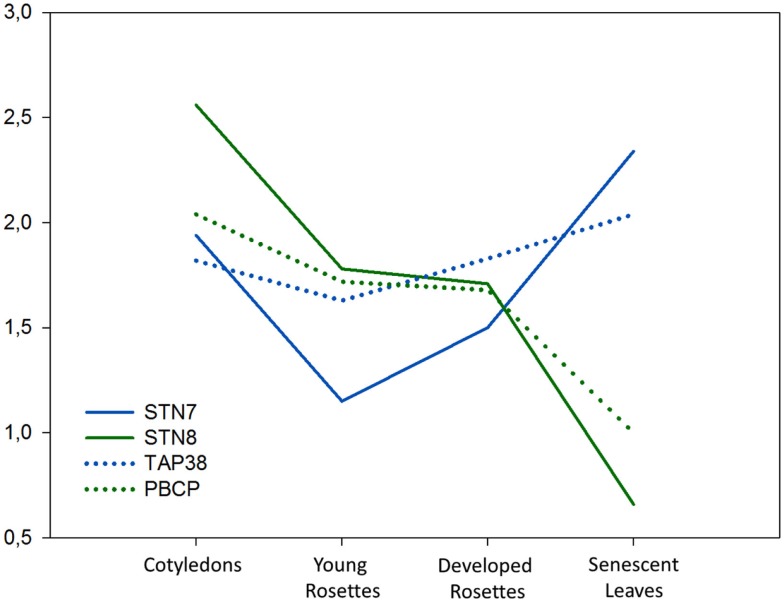
**Expression of *STN7*, *STN8*, *TAP38*, and *PBCP* in vegetative tissues during *Arabidopsis* development**. Expression data were extracted from microarray data sets ATGE_1_A–C (cotyledons), ATGE_22_A–C (young rosettes), ATGE_24_A–C (developed rosettes), and ATGE_25_A–C (senescent leaves) which are available in NCBI GEO database (http://www.ncbi.nlm.nih.gov/geo/). These arrays were analyzed in the curated Bio-Array Resource database (http://bar.utoronto.ca/). Expression level >1 shows upregulation, <1 shows downregulation compared to the median level of *STN7*, *STN8*, *TAP38*, or *PBCP* expression in the AtGeneExpress database (set as 1).

We analyzed the expression levels of *STN7*, *STN8*, *TAP38*, and *PBCP* genes in green tissues of *Arabidopsis* Columbia-0 at different stages of plant development using publicly available microarray data (Figure 3). Comparison of these data revealed higher expression of all four genes in cotyledons than in vegetative rosettes, and also closely correlated expression of respective kinase-phosphatase pairs (STN7-TAP38 and STN8-PCBP), which is in line with their shared substrates and likely antagonistic roles. Strikingly, the developmental expression profiles show clear upregulation of *STN7* and *TAP38* transcripts in senescent leaves, while the expression of *STN8* and *PBCP* can be seen to decline sharply (Figure 3). An identical trend was observed in rice microarrays (not shown). We observed a clear upregulation of *STN7* expression in response to numerous abiotic and biotic stress conditions which, along with the relatively high *STN7/TAP38* expression during plant senescence, appears to be in conflict with the observation that LHCII phosphorylation is low under such conditions of acceptor side limited photosynthesis (Rintamäki et al., 2000; Pursiheimo et al., 2001; Hou et al., 2003). Furthermore, a decrease in LHCII protein content that would be expected to occur under these conditions would lead to degradation of STN7 (Lemeille et al., 2009; Willig et al., 2011). One explanation for *STN7* upregulation during stress and senescence may be to compensate for an unavoidable degree of stress-related inactivation or degradation of STN7 in an attempt to maintain sufficient cellular levels of the kinase. It may also be possible that STN7 performs an alternative role in these conditions. A low chlorophyll a/b ratio in the *stn7* mutant, shown to be independent of thylakoid protein stoichiometry (Tikkanen et al., 2006), suggests a role for STN7 in either chlorophyll a synthesis or chlorophyll b degradation. Interestingly, our microarray analysis showed correlation between the expression profiles of *STN7* and several genes directly involved in isoprenoid biosynthesis and/or degradation, supporting a role in regulating chlorophyll accumulation. A final possibility may be that STN7/TAP38 activity is involved in the controlled degradation of LCHII, a process that is tightly regulated to prevent untimely release of phytotoxic chlorophyll which generates ROS (Kariola et al., 2005). Notably, chlorophyll b detoxification involves initial conversion to chlorophyll a (Hortensteiner and Krautler, 2011), which could explain the low chlorophyll a/b ratio in *stn7* that may be attributed to an inability to process chlorophyll b in the mutant. Furthermore, chlorophyll degradation products are known to operate in ROS-mediated retrograde signaling during plant stress response, but many other ROS signaling pathways exist, explaining the acquisition of a compensatory signaling mechanism in *stn7* that nonetheless became insufficient or ineffective under highly stressful conditions. STN7 regulation of LHCII decomposition and chlorophyll degradation would be particularly relevant under stress and during senescence, but may also be a mechanism for retrograde regulation of gene expression that is constitutively activated throughout plant development in response to the redox condition of the thylakoid and consequently also of the chloroplast stroma.

Unlike *STN7* and *TAP38*, *STN8* and *PCPB* expression levels decreased during senescence and were unaffected by biotic and abiotic stresses. The expression profiles *STN8* and *PCPB* correlated with a large number of photosystem subunits and factors regulating the expression of plastid genes and other chloroplast development processes, suggesting a primary role in regulating the biogenesis and function of photosynthetic membranes. It is interesting to note that *STN8*/*PCPB* expression was consistent during early and later stages of vegetative growth, while *STN7*/*TAP38* expression appeared to be higher in developed rosettes. Preliminary results from our lab show that the steady-state levels of LHCII phosphorylation regulate formation of PSII-LHCII-PSI-LHCI megacomplexes that are an important factor in lateral heterogeneity of photosynthetic membranes (Rantala and Aro, unpublished), which occurs only at later stages of plant development. Strong expression of the thylakoid kinases and phosphatases, particularly STN8, in cotyledons may relate to their importance in assembly of PSII during early plant development. Additionally, they may be active in customizing the photosynthetic machinery for effective utilization of light energy during these early stages when the seedling is not capable of handling large amounts of reducing power. In light of this conceivably vital role during early plant development, it is puzzling that the *stn8* and *stn7/stn8* mutants lack any seedling phenotype compared to WT plants, although it is now clear that STN8 activity becomes important at moderate high light intensities (500–1,000 μE m^−2^ s^−1^) that occur in nature but are lower than the light intensities commonly used in laboratory high light experiments. Interestingly, recent analysis of the *stn8* mutation in rice has described a stunted growth phenotype with severe problems in the degradation of damaged D1 protein (a congress poster and personal communication Choon-Hwan Lee and Krishna Nath).

## Redox State of Thylakoid Electron Transfer Chain Affects ROS Signaling in Plant Stress Response

**Figure 4:**
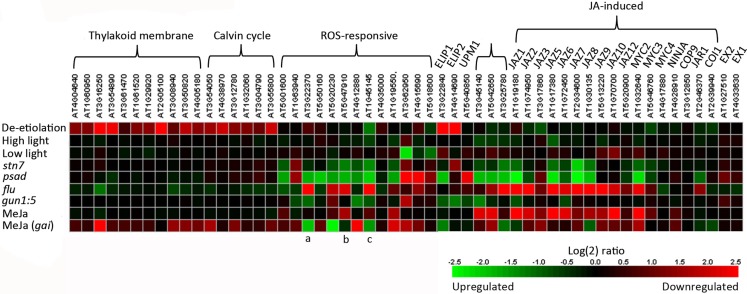
**Heat map comparing expression of relevant genes using microarray data available in the Genevestigator database (Hruz et al., 2008)**. Details of genes included can be found in Appendix. Light conditions shown are de-etiolation (120 min illumination of dark-adapted plants), high light treatment (120 min)/growth light, and low light (120 min)/growth light. Expression profiles of *stn7* (GEO ID: GSM399900 – 902) and *psad* (GEO ID: GSM99903 – 904) have interrupted thylakoid membrane redox balance, *flu* (GEO ID: GSM272987 – 988) constitutively produces singlet oxygen and *gun1:5* (GEO ID: GSM142773, 777, 779, 781) lacks chloroplast-to-nucleus signaling. Expression in methyl jasmonate (MeJa)-treated wildtype and giberellin-insensitive (*gai*) plants (Array Express ID: E-MEXP-883) is also shown. Specific ROS-responsive genes discussed in text are (a) AOX1a, (b) RbohD, and (c) TRX5.

We analyzed gene expression using the microarrays of *stn7* and *psad* mutants (Ihnatowicz et al., 2004, 2008; Pesaresi et al., 2009) to explore the effects of interrupted redox balance, caused by over reduction of the ETC between PSII and PSI, on the expression of thylakoid membrane, Calvin cycle, and ROS-responsive genes (Figure 4). We compared the expression profile of the *stn7* kinase mutant to the expression profiles of other mutants and paid special attention to mutants with disturbed thylakoid redox balance (*psad*; Pesaresi et al., 2009) or those devoid of supposedly essential components of retrograde signaling. The “genomes uncoupled” (*gun*) mutants have been demonstrated to have inhibited chloroplast-to-nucleus signaling (Koussevitzky et al., 2007) and the “conditional fluorescence” (*flu*) mutant was included to elucidate chloroplast ROS-initiated and EXECUTER-protein-mediated signaling from the chloroplast (Meskauskiene et al., 2001; op den Camp et al., 2003; Lee et al., 2007). These microarray data demonstrated that the expression of photosynthetic light harvesting and Calvin cycle genes was hardly affected by changes in ETC redox balance in *stn7* and *psad*, which is in line with earlier results from our lab showing these genes to be regulated by stromal or metabolic cues resulting from normal photosynthetic activity rather than by membrane redox signals (Fey et al., 2005; Piippo et al., 2006). On the other hand, *stn7* and *psad* knockout have a strong effect on the expression of various ROS-responsive genes (discussed below). Furthermore the regulation of these genes was similar in both mutants (Figure 4), indicating that both generate similar retrograde signals due to their shared perturbation to thylakoid membrane redox homeostasis.

Increasing evidence suggests that photosynthetic electron transfer reactions elicit ROS signals that mediate key functions in both abiotic and biotic stress responses in plants (Kangasjärvi et al., 2012; Suzuki et al., 2012). Recently, Nomura et al. (2012) demonstrated that the thylakoid CAS protein is required for suppression of photosynthesis-related genes and upregulation of pathogenesis-related genes upon recognition of bacterial flagellin in the extra-cellular space. Analysis of microarray profiles further revealed that one third of the genes with reduced induction in *cas-1* mutants overlapped with genes induced by singlet oxygen (^1^O_2_) in the *flu* system. In *flu* mutants, dark-induced accumulation of chlorophyll intermediates causes a massive release of singlet oxygen upon re-illumination, inducing strong expression of a specific set of ROS-responsive genes (op den Camp et al., 2003; Laloi et al., 2007). Thus, among thylakoid-associated pathways, CAS is required for full elicitation of ^1^O_2_-responsive gene expression changes under stress conditions, yet the role of CAS phosphorylation by STN8 in this process remains elusive.

To assess the capacities of *stn7* and *psad* mutant plants for generating ROS signals, we compared gene expression in these redox mutants with those in the *flu* mutant. Several genes that showed strongly upregulated expression in *flu* were strongly downregulated in the redox mutants, including the plasma membrane NADPH oxidase *RBOHD*, the mitochondrial alternative oxidase *AOX1a*, and the cytoplasmic thioredoxin *TRX5* (Figure 4), each of which mediates key functions in cellular redox signaling and is a major player in the regulation of plant immunity (Torres et al., 2002; Tada et al., 2008; Zhang et al., 2012). This apparently opposite regulation of ROS-induced gene expression in *stn7* and *psad* as compared to *flu* clearly demonstrates perturbed ROS signaling in the redox mutants. It is possible that altered PSI excitation due to ETC over reduction in *stn7* and *psad* (Pesaresi et al., 2009) limits the capacity to generate singlet oxygen that is normally produced by photosynthetic electron transfer, which may attenuate ROS signaling. Conceivably, alternative signaling pathways also contribute to the expression of ROS-responsive genes in the nucleus. Indeed, both *stn7* and *psad* show upregulation of the gene encoding chloroplast antioxidant enzyme *MDAR6*, which is correspondingly downregulated in *flu*. These signaling effects may be related to H_2_O_2_, which has been shown to act antagonistically to singlet oxygen-dependent signals (Laloi et al., 2007).

NADPH oxidases are key enzymes that promote pathogen or ozone induced ROS burst in the apoplast (Kangasjärvi et al., 2005). The downregulation of *RBOHD* occurring in *stn7* and *psad* suggests that over reduction of the intersystem ETC leads to an inhibited capacity to mediate ROS signals that originate from both inside and outside the chloroplast. This may further explain the lack of any obvious ROS-related stress phenotype in the redox mutants *stn7* and *psad* that would typically arise from uncontrolled ROS signals between the chloroplast and the rest of the cell. More broadly, these findings clearly connect redox homeostasis in the thylakoid membrane, as maintained by regulated excitation energy transfer between PSI and PSII, with the transmission of ROS signals throughout the plant cell. These analyses also support the view that the redox condition of the thylakoid membrane that is sensitive to various environmental cues instigates a cell-wide response, rather than specifically regulating the expression of photosynthesis-related genes.

## The Role of Membrane Protein Phosphorylation in Signaling Pathways within and Outside of the Chloroplast

**Figure 5:**
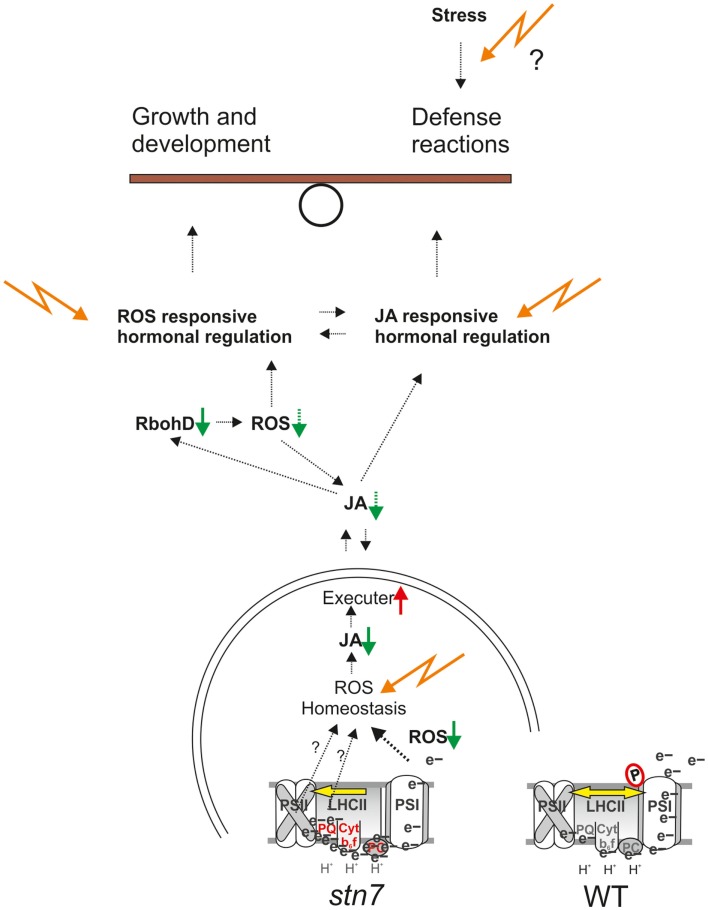
**A plant’s allocation of resources to growth or defense responses is determined by a balance between growth-promoting and the defense-promoting signaling pathways**. As illustrated in this model, we propose that regulation of the excitation energy distribution between PSII and PSI by thylakoid membrane protein phosphorylation controls cell signaling and systemic responses via ROS-induced, hormone-mediated signaling networks.

Microarray data show that cell-wide ROS signaling is sensitive to the accumulation of reducing power in ETC and the inability to reduce PSI electron acceptors; however it remains unclear how these signals are transmitted from the thylakoid membrane to the nucleus. Our analysis of gene expression in *stn7* and *psad* with disrupted redox balance showed generally downregulated expression of genes directly involved in the biosynthesis and regulation of jasmonic acid (JA), while the same genes are upregulated in high light and strongly upregulated in *flu* (Figure 4). The JA signaling pathway is induced in the chloroplasts of plants subjected to high light, biotic or abiotic stress, or any factors that limit plant metabolism and/or generate surplus reducing power that exceeds the capacity of stromal metabolism. JA signaling leads to downsizing of photosynthetic pigment-protein complexes at the level of gene expression (Figure 4), in order to downregulate the photosynthetic activity and alleviate the pressure on the ETC in stressed plants. The response to JA is clear in the microarray data, showing photosynthetic and Calvin cycle genes to be slightly or moderately downregulated (see Figure 4 and Appendix for details) during treatment with methyl jasmonate (MeJA), a derivative of JA. The same inhibitory effect on these genes is evident in the ROS-producing *flu* mutant, wherein the JA signaling pathway is clearly induced (Figure 4, Danon et al., 2005). JA synthesis enzymes and JA-induced genes encoding the MYC2 transcription factor and the “jasmonate-ZIM-domain” (JAZ) proteins are strongly downregulated in *stn7* and *psad*. This illustrates an absence of JA synthesis that occurs concomitantly with disturbed redox balance and inhibited ROS signaling in the redox mutants. Together these results suggest that JA, the biosynthesis of which is triggered by oxidation of thylakoid membrane fatty acids in the chloroplast, is a candidate for the missing intermediate in the redox-mediated signaling cascade. This aligns well with the observation that *RBOHD* is upregulated by JA treatment and downregulated in *psad* and *stn7* (Figure 4). ROS signals are transmitted from *flu* chloroplasts via the plastid EXECUTER proteins (EX1 and EX2; Lee et al., 2007), both of which are downregulated in the *flu* microarray (−1.2- and −1.4-fold, respectively) and upregulated in *psad* (2.1- and 1.5-fold, respectively) and *stn7* (1.3- and 1.1-fold, respectively). Based on the findings described above, we suggest that the redox state of the thylakoid membrane determines ROS production, which in turn induces oxylipin signals that are transmitted by EXECUTERs out of the chloroplast (see Figure 5).

Based on these observations, one could expect that the downregulation of JA signaling found in the *psad* and *stn7* mutants would lead to overexpression of the photosynthetic genes and enhanced growth of mutants. Indeed, the importance of JA signaling involves a role in balancing the stress response mechanisms with plant growth, to achieve optimum growth without compromising the stress tolerance. This occurs in collaboration between the JA and gibberellin (GA) signaling pathways (Yang et al., 2012). This cross-talk is clearly important for regulation of the expression of thylakoid membrane and Calvin cycle proteins, which are upregulated in MeJA-treated GA-insensitive (*gai*) mutant compared to MeJA-treated WT (Figure 4). The *stn7* mutant, however, grows normally, and the expression of photosynthetic genes is unchanged (Figure 4), suggesting that additional mechanisms can also regulate the expression of photosynthetic genes and/or compensate for interrupted JA synthesis. The *tap38* mutant, however, does grow faster than the WT under low light (Pribil et al., 2010). This phenotype could stem from a high level of LHCII phosphorylation and the subsequent decrease in PSII excitation in *tap38*, which would result in an inability to efficiently reduce PSI electron acceptors and therefore lead to a highly oxidized ETC. In this respect *tap38*, *stn7*, and *psad* share dysfunctional PSI, and so it is likely that *tap38* would also have decreased JA signaling from the chloroplast. The difference lies in the ETC redox states, namely over-reduced in *stn7* and *psad* and over-oxidized in *tap38*, which could induce differing signaling cascades that may explain their different growth phenotypes. Unfortunately no large-scale gene expression data from the *tap38* mutant is currently available, but it may be hypothesized from the evidence provided in this work that the redox state of the PQ pool and the ROS status of PSI operate in relaying information from the chloroplast to the cellular signaling networks for the control of nuclear gene expression.

## Concluding Remarks

An involvement of thylakoid kinases and phosphatases in regulating nuclear gene expression correlates with their relatedness to conventional signaling enzymes, and yet the unusually high concentration of phosphorylated proteins in photosynthetic membranes suggests an atypical function that may encompass a structure-dependent regulation of thylakoid activities. Our analysis indicates that a direct involvement of STN7 and membrane protein phosphorylation in signaling photosynthetic gene expression has been somewhat over-stated, and that retrograde signaling is more likely to be sensitive to the redox homeostasis of the membrane, which relies on the cooperative functioning of all photosynthetic components and their regulators. The mechanism for transmission of redox signals from the membrane to nucleus remains unclear; however, as illustrated in Figure 5, our results show that redox-sensitive production of ROS molecules is important for jasmonate synthesis in the chloroplast, and for hormone signaling in the wider cell.

In *stn7* and in other mutants with increased PSII function relative to PSI, such as *psad*, a decreased capacity the intersystem ETC is over-reduced while the PSI acceptor side is under-reduced, decreasing the capacity of PSI to produce H_2_O_2_ and possibly increasing ^1^O_2_ production by PSII and LHCII, thereby modifying ROS homeostasis of the thylakoid membrane. The altered ROS homeostasis downregulates the jasmonate (JA) biosynthesis pathway and upregulates the EXECUTER (EX1 and EX2) proteins proposed to mediate emission of ROS signals from the chloroplast. We propose that the signaling molecules mediated by EX1 and EX2 proteins are jasmonates produced from thylakoid lipids according to the photosynthetic ROS status, by the enzymes that are strongly downregulated in *stn7* and *psad* (Figure 4). Disrupted ROS homeostasis and subsequent reduction in JA synthesis can explain the downregulation of the plasma membrane NADPH oxidase RbohD, a JA-responsive ROS-producing enzyme at the center of ROS- and hormone-mediated networks of plant defense response.

Reactive oxygen species signals impact hormone signaling pathways that operate at all levels of cellular metabolism, both through direct interaction and via JA. Under standard growth conditions, the effects of ETC redox imbalance on hormone regulation appear to be buffered or counteracted by overlapping and redundant signaling pathways. It is unknown, however, how these readjustments to redox imbalance may compromise a plant’s ability to defend against plant pathogens, which is particularly relevant in the natural environment that presents continuous fluctuations in environmental conditions and an array of other abiotic and biotic stresses.

## Conflict of Interest Statement

The authors declare that the research was conducted in the absence of any commercial or financial relationships that could be construed as a potential conflict of interest.

## Appendix

**Table:**

Function	Localization	AGI locus identifier	Description	Expression fold change	Treatment
Photosynthesis	Thylakoid membrane	At4g04640	ATP synthase subunit gamma	−1.11	MeJA
		At1g60950	Ferredoxin A	−1.02	
		At3g16250	Photosynthetic NDH subcomplex B3	−1.35	
		At3g54890	PSI light harvesting protein Lhca1	−1.06	
		At3g61470	PSI light harvesting protein Lhca2	−1.14	
		At1g61520	PSI light harvesting protein Lhca3	−1.06	
		At1g29930	PSII chlorophyll a/b-binding protein Lhcb1.3	−1.16	
		At2g05070	PSII chlorophyll a/b-binding protein Lhcb2.2	−1.47	
		At3g08940	PSII chlorophyll a/b-binding protein Lhcb4.2	−1.20	
		At3g50820	PsbO subunit of PSII oxygen-evolving complex	−1.24	
		At4g05180	PsbQ subunit of PSII oxygen-evolving complex	−1.02	
Calvin cycle	Chloroplast stroma	At3g54050	Fructose 1,6-bisphosphate phosphatase	−1.62	MeJA
		At4g38970	Fructose 1,6-bisphosphate aldolase	−1.08	
		At3g12780	Phosphoglycerate kinase	−1.08	
		At1g32060	Phosphoribulokinase	−1.28	
		At3g04790	Ribose 5-phosphate isomerase	−1.18	
		At3g55800	Sedoheptulose bisphosphatase	−1.14	
ROS-responsive	Chloroplast	At5g01600	Ferritin 1		
		At1g63940	Monodehydroascorbate reductase 6 (MDAR6)	
	Mitochondrion	At3g22370	Alternative oxidase 1a (AOX1a)	
		At5g50160	Ferric chelate reductase	
	Plasma membrane	At5g20230	Copper-binding protein/Senescence-associated gene 14 (SAG14)	
		At5g47910	Respiratory burst oxidase homolog D (RboHD)	
	Apoplast	At4g12880	Early nodulin-like protein 19 (ENODL19)	
	Cytoplasm	At1g45145	Thioredoxin 5	
		At4g35000	Ascorbate peroxidase 3	
		At1g19570	Dehydroascorbate reductase 1/5	
		At5g18600	Glutaredoxin-like protein	
	ER	At3g62950	Glutaredoxin (GRXC11)	
		At4g15690	Glutaredoxin (GRXS5)	
Chlorophyll-binding	Thylakoid	At3g22840	Early light-inducible protein 1 (ELIP1)	
	Membrane	At4g14690	Early light-inducible protein 2 (ELIP2)	
Siroheme biosynthesis	Chloroplast	At5g40850	Uroporphyrin methyltransferase (UPM1)	
JA synthesis enzymes	Chloroplast	At3g45140	Lipoxygenase 2 (LOX2)	
		At5g42650	Allene oxide synthase	
		At3g25780	Allene oxide cyclize	
Negative regulation of JA-induced gene expression	Nucleus	At1g19180	Jasmonate ZIM-domain protein 1 (JAZ1)	
		At1g74950	Jasmonate ZIM-domain protein 2 (JAZ2)	
		At3g17860	Jasmonate ZIM-domain protein 3 (JAZ3)	
		At1g17380	Jasmonate ZIM-domain protein 5 (JAZ5)	
		At1g72450	Jasmonate ZIM-domain protein 6 (JAZ6)	
		At2g34600	Jasmonate ZIM-domain protein 7 (JAZ7)	
		At1g30135	Jasmonate ZIM-domain protein 8 (JAZ8)	
		At1g70700	Jasmonate ZIM-domain protein 9 (JAZ9)	
		At5g13220	Jasmonate ZIM-domain protein 10 (JAZ10)	
		At5g20900	Jasmonate ZIM-domain protein 12 (JAZ12)	
		At4g28910	Novel interactor of JAZ (NINJA)	
Transcriptional activator of JA-responsive gene expression	Nucleus	At1g32640	MYC2	
		At5g46760	MYC3	
		At4g17880	MYC4	
JA signaling regulation	Cytoplasm/nucleus	At3g12850	Involved in COP9 signalosome complex	
Catalyzes JA-Ile synthesis, promotes JA signaling pathway	Cytoplasm	At2g46370	JA resistance1 (JAR1)	
Interacts with JAZ, required for JA-induced gene expression	Nucleus	At2g39940	Coronatine insensitive 1 (COI1)	
Relay of singlet oxygen-induced signal	Thylakoid	At4g33630	Executer1 (EX1)	
	Membrane	At1g27510	Executer2 (EX2)	

## Abbreviations

ETC, electron transfer chain; JA, jasmonic acid; LHC, light harvesting complex; LTR, long-term response; MeJA, methyl jasmonate; PC, plastocyanin; PQ, plastoquinone; PS, photosystem; ROS, reactive oxygen species.
